# Evaluation of Stillbirth Among Pregnant People With Sickle Cell Trait

**DOI:** 10.1001/jamanetworkopen.2021.34274

**Published:** 2021-11-24

**Authors:** Silvia P. Canelón, Samantha Butts, Mary Regina Boland

**Affiliations:** 1 Department of Biostatistics, Epidemiology and Informatics, University of Pennsylvania, Philadelphia; 2 Division of Reproductive Endocrinology and Infertility, Penn State College of Medicine and Penn State Health, Hershey, Pennsylvania; 3 Institute for Biomedical Informatics, University of Pennsylvania, Philadelphia; 4 Center for Excellence in Environmental Toxicology, University of Pennsylvania, Philadelphia; 5 Department of Biomedical and Health Informatics, Children’s Hospital of Philadelphia, Philadelphia, Pennsylvania

## Abstract

**Importance:**

Relative to what is known about pregnancy complications and sickle cell disease (SCD), little is known about the risk of pregnancy complications among those with sickle cell trait (SCT). There is a lack of clinical research among sickle cell carriers largely due to low sample sizes and disparities in research funding.

**Objective:**

To evaluate whether there is an association between SCT and a stillbirth outcome.

**Design, Setting, and Participants:**

This retrospective cohort study included data on deliveries occurring between January 1, 2010, and August 15, 2017, at 4 quaternary academic medical centers within the Penn Medicine health system in Pennsylvania. The population included a total of 2482 deliveries from 1904 patients with SCT but not SCD, and 215 deliveries from 164 patients with SCD. Data were analyzed from May 3, 2019, to September 16, 2021.

**Exposures:**

The primary exposure of interest was SCT, identified using clinical diagnosis codes recorded in the electronic health record.

**Main Outcomes and Measures:**

A multivariate logistic regression model was constructed to assess the risk of stillbirth using the following risk factors: SCD, numbers of pain crises and blood transfusions before delivery, delivery episode (as a proxy for parity), prior cesarean delivery, multiple gestation, patient age, marital status, race and ethnicity, ABO blood type, Rhesus (Rh) factor, and year of delivery.

**Results:**

This cohort study included 50 560 patients (63 334 deliveries), most of whom were aged 25 to 34 years (29 387 of 50 560 [58.1%]; mean [SD] age, 29.5 [6.1] years), were single at the time of delivery (28 186 [55.8%]), were Black or African American (23 777 [47.0%]), had ABO blood type O (22 879 [45.2%]), and were Rhesus factor positive (44 000 [87.0%]). From this general population, 2068 patients (4.1%) with a sickle cell gene variation were identified: 1904 patients (92.1%) with SCT (2482 deliveries) and 164 patients (7.9%) with SCD (215 deliveries). In the fully adjusted model, SCT was associated with an increased risk of stillbirth (adjusted odds ratio [aOR], 8.94; 95% CI, 1.05-75.79; *P* = .045) while adjusting for the risk factors of SCD (aOR, 26.40; 95% CI, 2.48-280.90; *P* = .007) and multiple gestation (aOR, 4.68; 95% CI, 3.48-6.29; *P* < .001).

**Conclusions and Relevance:**

The results of this large, retrospective cohort study indicate an increased risk of stillbirth among pregnant people with SCT. These findings underscore the need for additional risk assessment during pregnancy for sickle cell carriers.

## Key Points

**Question:**

Is sickle cell trait associated with an increased risk of stillbirth?

**Findings:**

In this cohort study of 50 560 patients and 63 334 deliveries (including 2482 deliveries from patients with sickle cell trait and 215 deliveries from patients with sickle cell disease) both sickle cell trait and sickle cell disease were associated with increased risk of stillbirth after adjusting for known risk factors.

**Meaning:**

This study found both sickle cell trait and disease to be associated with an increased risk of stillbirth, suggesting that sickle cell carriers would benefit from additional risk assessment during pregnancy.

## Teaser

This cohort study investigates the association between sickle cell trait and risk of stillbirth in pregnant people.

## Introduction

Sickle cell disease (SCD) is a severe and complex inherited genetic disorder and the most common hemoglobinopathy in the US, currently affecting roughly 100 000 individuals.^1,2^ Individuals with 1 abnormal allele of the hemoglobin gene (HbS, heterozygous) have sickle cell trait (SCT), whereas those with 2 abnormal alleles have SCD. Sickle cell disease is particularly prevalent in those of African ancestry because of the protective effects of SCT against malaria^3,4,5^ and is associated with high lifetime morbidity and premature mortality,^6^ particularly as a result of chronic complications.^7^ Sickle cell trait is not considered a disease state because many sickle cell carriers have at least 50% normal adult hemoglobin^8^ and are asymptomatic.^9,10^ However, it is possible for people with SCT to experience sickling of red blood cells under severe hypoxia, dehydration, and hyperthermia. This condition can lead to severe medical complications for sickle cell carriers, including fetal loss,^11^ splenic infarction, exercise-related sudden death, and others.^12^

Individuals affected by SCD in the US often lack access to specialized care and comprehensive primary care compared with those with less common hematologic disorders, such as hemophilia, or other genetic diseases that primarily afflict those of European ancestry, such as cystic fibrosis.^13^ Genetic diseases that predominantly affect European ancestry populations often have extensive networks of comprehensive and multidisciplinary specialty care centers enabling the creation and use of patient registries to monitor outcomes and evaluate the effectiveness of treatments.^13^ This stands in sharp contrast with the care available to patients with SCD, whose access to subspecialty care is much more fragmented.^13,14^ This disparity is also reflected by funding mechanisms that disproportionately favor diseases of European ancestry over SCD despite SCD having a higher incidence in the US.^15,16,17^ These disparities affect all aspects of health care and research, dating back to 1970 and persisting today.^16^

Individuals who are pregnant and living with SCD are at an increased risk of complications.^6,18,19,20,21,22^ In contrast, the existing evidence for pregnant patients with SCT is sparse and, at most, points to probable or moderate associations between SCT and complications such as preeclampsia, prematurity, or fetal loss or death.^12,23^

It can be difficult to determine whether these pregnancy-related complications are due to SCT, SCD, and/or health disparities common among Black or African American individuals^24,25^ resulting from structural racism.^26,27,28,29^ Our study explores these interactions by assessing the association of sickle cell status and race and ethnicity on the risk of pregnancy-related complications by leveraging electronic health records (EHRs) within a large hospital system. Electronic health records contain rich information on patient medical history, including records detailing sickle cell encounters,^30,31,32^ and can be used to study associations between SCT and pregnancy-related complications such as stillbirth.

## Methods

**Figure 1.**
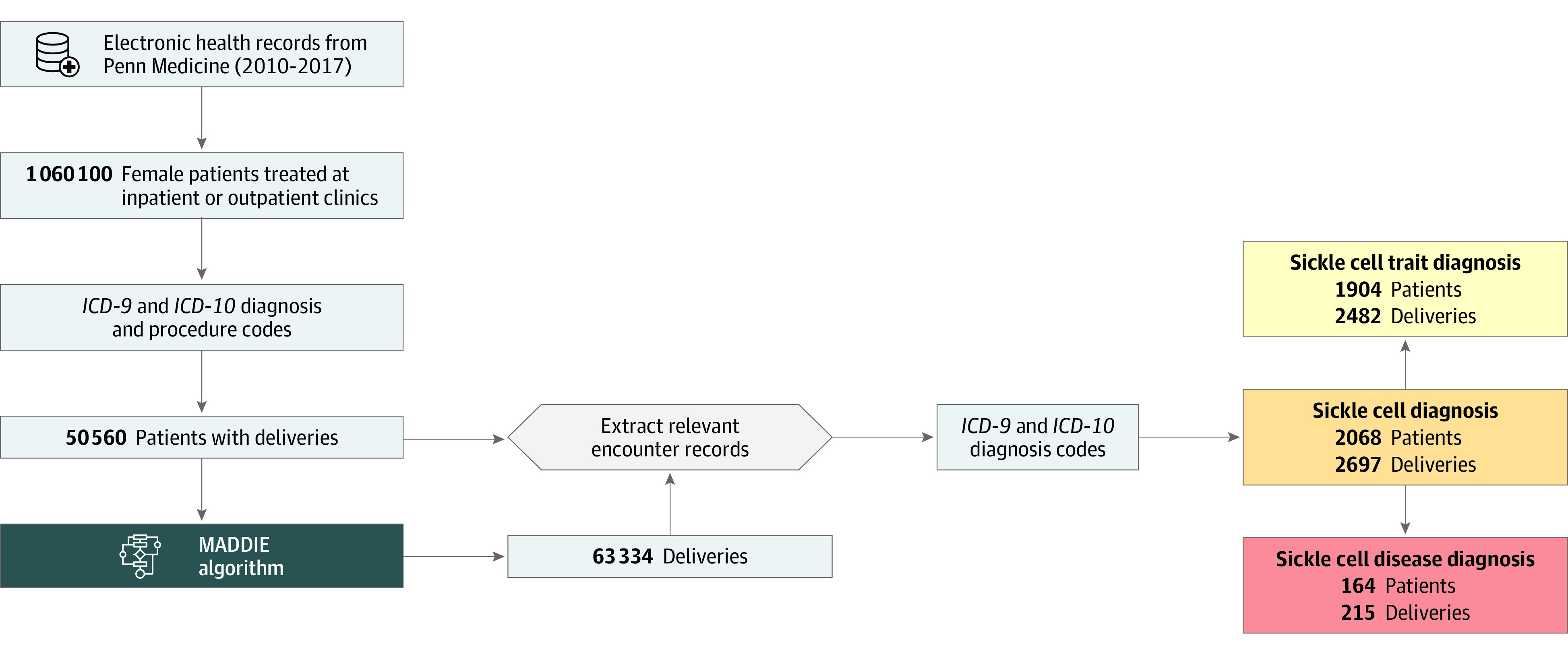
Data Analysis Pipeline From the Penn Medicine Database to the Identification of Patients With Deliveries and Sickle Cell Diagnoses The Method to Acquire Delivery Date Information From Electronic Health Records (MADDIE) algorithm identified 63 334 deliveries from 50 560 patients with deliveries at Penn Medicine between 2010 and 2017. Relevant encounter records were extracted for these patients and their delivery encounters, and *International Classification of Diseases, Ninth Revision *(*ICD-9*) and *International Statistical Classification of Diseases and Related Health Problems, Tenth Revision* (*ICD-10*) codes were used to identify patients with a sickle cell trait diagnosis or a sickle cell disease diagnosis. Patients in either of these groups were categorized more broadly as patients with a sickle cell gene variation.

This retrospective cohort study leveraged structured EHR data to study stillbirths among delivering patients with SCT at Penn Medicine in Pennsylvania between January 1, 2010, and August 15, 2017. The EHR data originated from 4 hospitals and contained a combination of inpatient and outpatient encounters with details including diagnosis and procedure billing codes along with patient demographic details at the time of the encounter. A total of 63 334 deliveries were identified using a previously developed algorithm that extracts delivery episode details and delivery dates from the EHR.^33^ These delivery episode details allowed us to (1) identify complications present for each of a patient’s deliveries and (2) examine the association of SCT^24^ with a patient’s likelihood of experiencing a stillbirth. We linked this information with encounter-level information on patient age and marital status at the time of the encounter, self-reported race and ethnicity, ABO blood type, and Rhesus factor (Figure 1). The EHR data were stored in a MySQL database on a Health Insurance Portability and Accountability Act–secure server. This study was approved by the institutional review board of the University of Pennsylvania. An exemption of consent was granted due to retrospective analysis of existing clinical records. This study followed the Strengthening the Reporting of Observational Studies in Epidemiology (STROBE) reporting guideline.

### Directed Acyclic Graph

**Figure 2.**
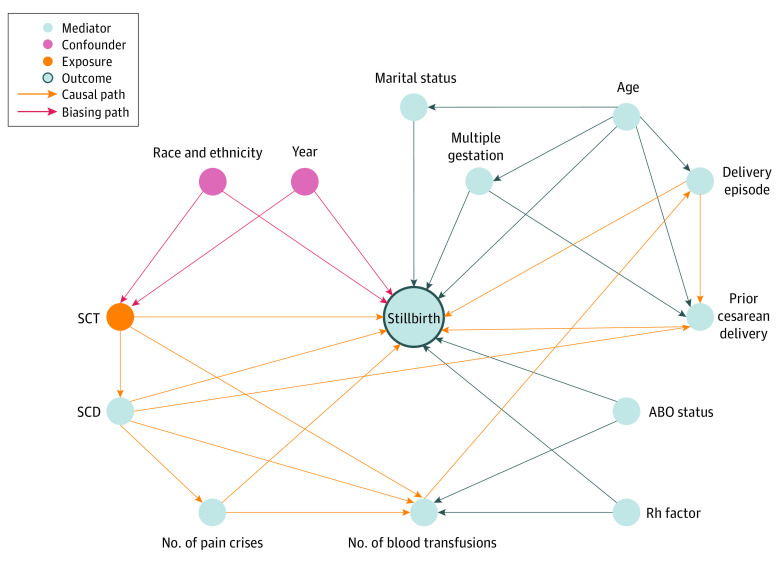
Directed Acyclic Graph With Stillbirth as the Outcome and Sickle Cell Trait (SCT) as the Primary Exposure of Interest Confounders include race and ethnicity and year, and biasing paths connect each of these 2 variables with SCT and stillbirth. Causal paths connect SCT with stillbirth by way of various mediators. Mediators include sickle cell disease (SCD), the number of pain crises before delivery, the number of blood transfusions before delivery, ABO blood type, Rhesus factor, marital status, age, multiple gestation, and delivery episode.

We hypothesized that SCT would be associated with stillbirth and created a directed acyclic graph to illustrate our baseline assumptions based on collective domain expertise and associations reported in the literature (Figure 2). The minimal sufficient adjustment sets for estimating the association of SCT with stillbirth were the observed confounders of race and ethnicity and year. Any variables corresponding to these confounders were included in the regression model. Mediators include SCD, the number of pain crises before delivery, the number of blood transfusions before delivery, ABO blood type, Rhesus factor, marital status, age, multiple gestation, and delivery episode. Variables corresponding to each of these mediators were also adjustments included in the regression model.

Given that SCT primarily affects people of African ancestry^3,4,5^ and that this same group of people has been found to be at risk of stillbirth,^34,35,36^ race and ethnicity were designated as a confounder. The year of the delivery and stillbirth outcome (when present) was designated as a confounder given that we observed variations in the rate of stillbirth over time (eFigure in the Supplement).

As illustrated in Figure 2, SCD is a successor of SCT because SCD requires 2 abnormal hemoglobin alleles and SCT requires 1. Sickle cell disease also connects to stillbirth because SCD has been associated with stillbirth in primary literature^21,37,38^ and meta-analyses.^18^ Sickle cell disease connects to the number of prior blood transfusions because transfusions are used in the management of SCD to prevent sickling crises.^8^ Additionally, blood transfusions are sometimes used prophylactically during pregnancy^39,40^ and are more common among pregnant people with SCD.^22^ For these reasons, we also connect blood transfusions to the number of prior pain crises. The latter descends from SCD for 2 primary reasons: pregnant people with SCD are at risk of pain crisis during delivery,^20,37^ and all patients diagnosed with pain crises in our study are also implicitly diagnosed with SCD in this study (eg, *International Classification of Diseases, Ninth Revision [ICD-9]* code 282.62 for sickle cell anemia is described as HbSS disease with crisis). Pain crises connect to stillbirth because of the effect sickling can have on the placenta and fetal viability.^41^ Other variables include ABO blood type, which has been associated with stillbirth,^35^ and Rhesus factor for the connection between Rh incompatibility and miscarriage.^42,43^

In our study, the delivery episode variable served as a proxy for parity and connects to stillbirth because parity has been associated with stillbirth in the literature.^34,36^ The presence of a prior cesarean delivery connects to stillbirth because it is a risk factor^34^ and has been associated with SCD.^6,20,21,22^

Patient age connects to prior cesarean deliveries and to the delivery episode with the understanding that as a patient gets older, they are more likely to have experienced a pregnancy and a cesarean section. For similar reasons, age connects to marital status, which was a binary variable for “married” in this study. Age has been associated with stillbirth,^34,35^ and being unmarried has been identified as a risk factor for stillbirth.^35^ Lastly, the relationship between age and multiple gestation is well-known,^44^ and multiple gestation has been associated with stillbirth.^35^

### Identification of Sickle Cell Status

We identified patients diagnosed with either SCT or SCD using *ICD-9* and *International Statistical Classification of Diseases and Related Health Problems, Tenth Revision* (*ICD-10*) codes. Patients diagnosed with SCD were considered patients with SCD, and those diagnosed specifically with the trait but not the disease were considered patients with SCT. Patients with SCD included those with sickle cell thalassemia (code 282.42), sickle cell anemia (code 282.62), sickle cell hemoglobin C disease (code 282.64), and other sickle cell disorders (eTable 1 in the Supplement). All patients with a sickle cell gene variation (SCD or SCT) were considered more broadly as “patients with sickle cell,” a grouping used in the regression model to adjust for any genetic inheritance of SC.

### Identification of Stillbirth and Other Complications

We used *ICD-9* and *ICD-10* codes to identify stillbirth outcomes. Clinical complications, including cesarean deliveries, multiple gestation, pain crises, and blood transfusions, were identified the same way and adjusted for in the regression model. Codes for pain crises and blood transfusions were obtained from the Centers for Disease Control and Prevention’s list of severe maternal morbidity indicators,^45^ and blood transfusion codes included red cell exchange procedures (eTable 1 in the Supplement). Codes for stillbirths, cesarean deliveries, and multiple gestation were obtained manually (eTable 1 in the Supplement).

### Statistical Analysis

We constructed a multivariate logistic regression model to explore the relationship between SCT and stillbirth as the outcome of interest. Sickle cell trait was an independent variable, and we adjusted for several variables hypothesized to alter the risk of stillbirth (Figure 2), with α = .05 defined as significant. A diagnosis of SCT was considered a binary independent variable in the model. Independent variables in our model included patient age as a continuous variable, and marital status as a binary variable, ie, married vs not married, both determined at time of delivery. All race and ethnicity variables were included as binary variables, eg, Asian vs non-Asian. All racial groups were non-Hispanic, with Hispanic ethnicity assessed separately. The decision to include race and ethnicity as independent variables has no biological basis but rather is grounded in an effort to explore how racism (structural or otherwise) may be reflected in stillbirth outcomes.

Delivery episode, multiple gestation, and prior cesarean delivery were also included as binary independent variables. The delivery episode was defined as the number of deliveries per patient at Penn Medicine within the 2010-2017 time frame. In this way, delivery episode acted a proxy for parity in our data set. In addition, a clinical complication code assigned within a patient’s delivery episode was used to link a stillbirth, cesarean delivery, multiple gestation, pain crisis, or blood transfusion event to a specific delivery or pregnancy. To account for any variability by year, we included year as a categorical independent variable in our model, including the years 2010 to 2017, with 2010 serving as the reference.

An SCD diagnosis was included as a binary independent variable. Because SCD is a chronic disease, often resulting in multiple blood transfusions over the course of a lifetime, red blood cell alloimmunization and blood type information are important to consider.^8,46,47^ We included ABO blood type and Rhesus factor as categorical independent variables and the number of prior blood transfusions as a continuous independent variable. The number of prior pain crises was included as a continuous independent variable (available only for patients with SCD).

Our fully adjusted model included each distinct race and ethnicity group because SCT and SCD relate to African ancestry, and Black or African American individuals are at increased risk of multiple delivery complications due to health disparities^24,25^ and structural racism.^26,27,48^

For comparison purposes, we also ran a regression model for stillbirth using a stratified data set of only Black or African American patients, given that most patients with SCT and SCD identified as Black or African American. This model did not include race and ethnicity variables because the stratified data set was uniform in this regard. All programming code for the data analysis and visualization was implemented in R, version 4.1.0 (R Foundation),^49^ using the tidyverse^50^ and forester packages.^51^ All *P* values were 2-sided. Data were analyzed from May 3, 2019, to September 16, 2021.

## Results

This cohort study included 50 560 patients (63 334 deliveries), most of whom were aged 25 to 34 years old (58.1%; mean [SD] age, 29.5 [6.1] years), were single at the time of delivery (55.8%), had ABO blood type O (45.2%), and were Rhesus factor positive (87.0%). Patient race and ethnicity included American Indian or Alaska Native (0.1%), Asian (6.5%), Black or African American (47.0%), Native Hawaiian or Pacific Islander (0.1%), White (33.7%), other or mixed (3.2%), and unknown (1.7%). The groupings of “other or mixed” reflected the structured data available in the EHR and not a custom grouping particular to this study. From this general population, 2068 patients with sickle cell were identified: 1904 patients with SCT (2482 deliveries) and 164 patients with SCD (215 deliveries). Black or African American patients accounted for 93.4% of patients with SCT and 87.2% with SCD (Table 1).

**Table 1.** Characteristics of Patients Within the Penn Medicine Cohort

Patient characteristics	No. (%)							
	Total population		Sickle cell trait		Sickle cell disease		Sickle cell gene variation	
	Patients	Deliveries	Patients	Deliveries	Patients	Deliveries	Patients	Deliveries
No. (%)	50 560 (100)	63 334 (100)	1904 (3.8)	2482 (3.9)	164 (0.3)	215 (0.3)	2068 (4.1)	2697 (4.3)
Patient age, y								
Mean (SD)	29.5 (6.1)	NA	NA	NA	NA	NA	NA	NA
<18^a^	1092 (2.2)	1117 (1.8)	81 (4.2)	82 (3.3)	NA^b^	NA^b^	85 (4.1)	86 (3.2)
18-24	11 053 (21.9)	12 661 (20.0)	687 (36.1)	790 (31.8)	54 (32.9)	62 (28.8)	741 (35.8)	852 (31.6)
25-34	29 387 (58.1)	34 799 (55.0)	964 (50.6)	1178 (47.5)	91 (55.5)	110 (51.2)	1055 (51.0)	1288 (47.8)
35-44	10 609 (21.0)	11 889 (18.8)	262 (13.8)	292 (11.8)	22 (13.4)	26 (12.1)	284 (13.7)	318 (11.8)
≥45	2858 (5.7)	2868 (4.5)	139 (7.3)	140 (5.6)	13 (7.9)	13 (6.0)	152 (7.3)	153 (5.7)
Marital status								
Single	28 186 (55.8)	34 823 (55.0)	1512 (79.4)	1950 (78.6)	120 (73.2)	154 (71.6)	1632 (78.9)	2104 (78.0)
Married	21 848 (43.2)	27 795 (43.9)	371 (19.5)	499 (20.1)	41 (25.0)	57 (26.5)	412 (19.9)	556 (20.6)
Separated, divorced, or widowed	568 (1.1)	672 (1.1)	28 (1.5)	33 (1.3)	3 (1.8)	4 (1.9)	31 (1.5)	37 (1.4)
Other or unknown	43 (0.1)	44 (0.1)	NA	NA	NA	NA	NA	NA
Race and ethnicity^c^								
American Indian or Alaska Native	61 (0.1)	81 (0.1)	NA	NA	NA	NA	NA	NA
Asian	3305 (6.5)	4073 (6.4)	6 (0.3)	7 (0.3)	5 (3.0)	7 (3.3)	11 (0.5)	14 (0.5)
Black or African American	23 777 (47.0)	29 965 (47.3)	1778 (93.4)	2331 (93.9)	143 (87.2)	187 (87.0)	1921 (92.9)	2518 (93.4)
Hispanic	4031 (8.0)	4985 (7.9)	49 (2.6)	57 (2.3)	NA^b^	5 (2.3)	53 (2.6)	62 (2.3)
Native Hawaiian or Pacific Islander	75 (0.1)	94 (0.1)	NA	NA	NA	NA	NA	NA
White	17 034 (33.7)	21 443 (33.9)	29 (1.5)	37 (1.5)	9 (5.5)	12 (5.6)	38 (1.8)	49 (1.8)
Other or mixed^d^	1644 (3.2)	2022 (3.2)	29 (1.5)	37 (1.5)	NA^b^	NA^b^	31 (1.5)	40 (1.5)
Unknown	865 (1.7)	971 (1.5)	18 (0.9)	18 (0.7)	NA^b^	NA^b^	21 (1.0)	22 (0.8)
Multiple birth	1555 (3.1)	1562 (2.5)	61 (3.2)	61 (2.5)	6 (3.7)	6 (2.8)	67 (3.2)	67 (2.5)
Blood type								
A	14 847 (29.4)	18 784 (29.7)	481 (25.3)	637 (25.7)	55 (33.5)	76 (35.4)	536 (25.9)	713 (26.4)
B	8396 (16.6)	10 649 (16.8)	392 (20.6)	523 (21.1)	34 (20.7)	45 (20.9)	426 (20.6)	568 (21.1)
AB	2209 (4.4)	2779 (4.4)	81 (4.2)	100 (4.0)	5 (3.0)	6 (2.8)	86 (4.2)	106 (3.9)
O	22 879 (45.2)	28 884 (45.6)	916 (48.1)	1188 (47.9)	68 (41.5)	86 (40.0)	984 (47.6)	1274 (47.2)
Unknown	2232 (4.4)	2238 (3.5)	34 (1.8)	34 (1.4)	NA^b^	NA^b^	36 (1.7)	36 (1.3)
Rhesus blood factor								
Positive	44 000 (87.0)	55 606 (87.8)	1763 (92.6)	2304 (92.8)	152 (92.7)	197 (91.6)	1915 (92.6)	2501 (92.7)
Negative	4331 (8.6)	5490 (8.7)	107 (5.6)	144 (5.8)	10 (6.1)	16 (7.4)	117 (5.7)	160 (5.9)
Unknown	2232 (4.4)	2238 (3.5)	34 (1.8)	34 (1.4)	NA^b^	NA^b^	36 (1.7)	36 (1.3)

Abbreviations: NA, not applicable.

^a^ Patients were 18 years or older at the time of the institutional review board approval but younger than 18 years at the time of delivery.

^b^ Patient and delivery counts smaller than 5 were omitted to preserve privacy.

^c^ Descriptions are non-Hispanic unless otherwise indicated.

^d^ The groupings of “Other and Mixed” reflect the structured data available in the EHR and not a custom grouping particular to this study.

**Figure 3.**
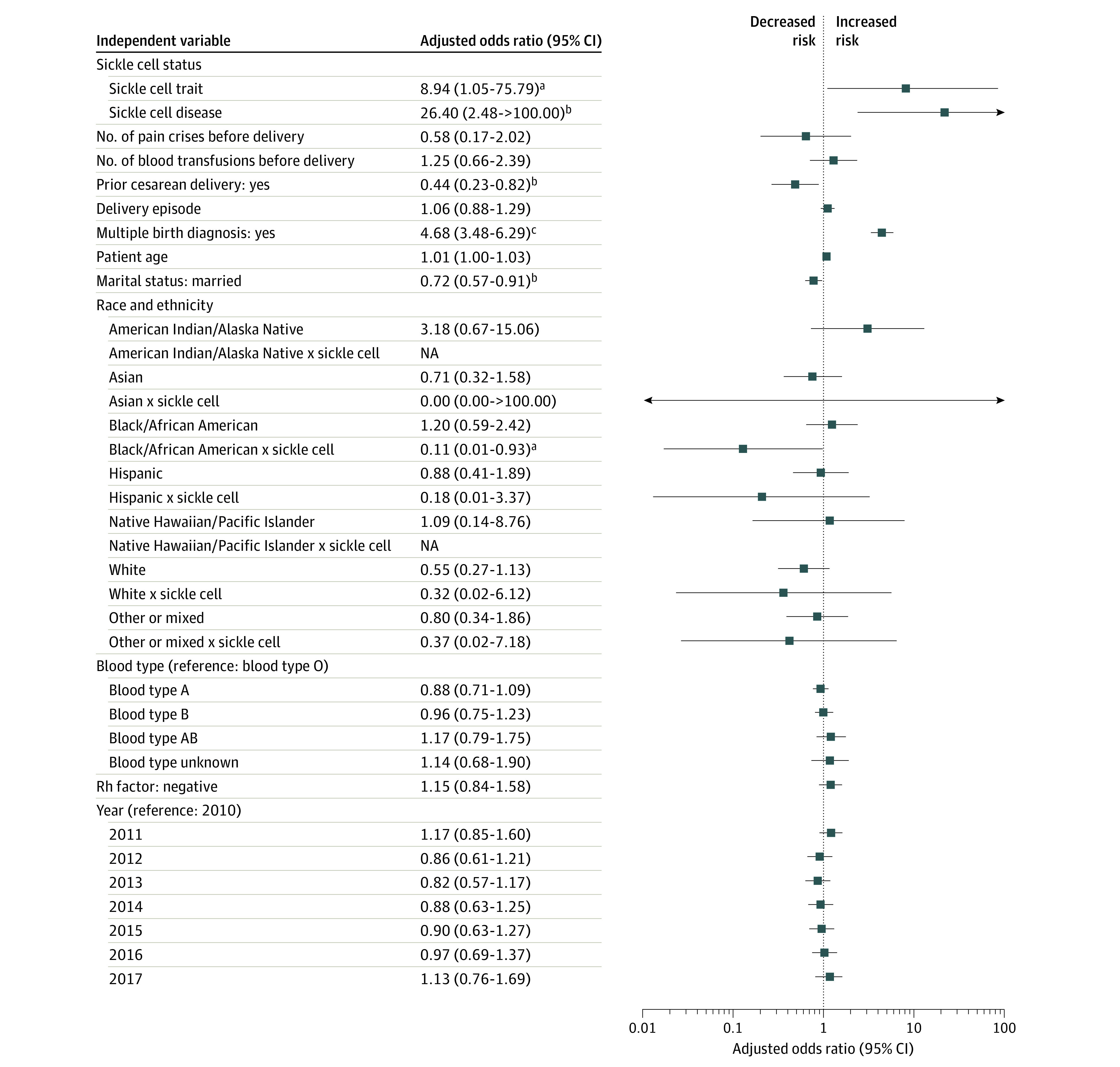
Estimated Association Between Patient Variables and Risk of Stillbirth Significant variables: Sickle cell trait (SCT), sickle cell disease (SCD), prior cesarean delivery, multiple gestation diagnosis, married marital status, and Black or African American race with sickle cell. Nonsignificant variables: number of pain crises before delivery, number of blood transfusions before delivery, delivery episode, patient age, Hispanic ethnicity, Hispanic ethnicity with sickle cell, Black or African American race, White race, White race with sickle cell, Asian race, Asian race with sickle cell, other or mixed race and ethnicity, other or mixed race and ethnicity with sickle cell, Native Hawaiian or Pacific Islander race, Native Hawaiian or Pacific Islander race with sickle cell, American Indian or Alaska Native race, American Indian or Alaska Native race with sickle cell, blood type A, blood type B, blood type AB, blood type unknown, Rhesus (Rh) factor negative, 2011, 2012, 2013, 2014, 2015, 2016, and 2017. Data presented in this figure can be found presented as a table in eTable 2 in the Supplement). The groupings of “other and mixed” reflect the structured data available in the EHR and not a custom grouping particular to this study. NA indicates not available. ^a^Indicates *P* < .05. ^b^Indicates *P* < .01. ^c^Indicates *P* < .001.

Of the total 63 334 deliveries, 516 (0.8%) resulted in a stillbirth (Table 2), and 2482 (3.9%) were by patients with SCT. The prevalence of stillbirth was 1.1% among patients with SCT compared with 0.8% in the general population (Table 2), and stillbirth was found to be associated with SCT after adjusting for all risk factors. In the fully adjusted model, patients with SCT were at higher risk of stillbirth relative to patients without SCT (adjusted odds ratio [aOR], 8.94; 95% CI, 1.05-75.79; *P* = .045) (Figure 3; eTable 2 in the Supplement).

**Table 2.** Stillbirth Rates Within the Penn Medicine Cohort

Patient population	No. (%)							
	Total population		Sickle cell trait		Sickle cell disease		Sickle cell gene variation	
	Patients	Deliveries	Patients	Deliveries	Patients	Deliveries	Patients	Deliveries
Total population	50 560 (100)	63 334 (100)	1904 (3.8)	2482 (3.9)	164 (0.3)	215 (0.3)	2068 (4.1)	2697 (4.3)
Stillbirth	507 (1.0)	516 (0.8)	26 (1.4)	27 (1.1)	5 (3.0)	5 (2.3)	31 (1.5)	32 (1.2)
Black or African American population	23 777 (100)	29 965 (100)	1778 (100)	2331 (100)	143 (100)	187 (100)	1921 (100)	2518 (100)
Stillbirth	323 (1.4)	329 (1.1)	22 (1.2)	23 (1.0)	5 (3.5)	5 (2.7)	27 (1.4)	28 (1.1)

Of the total 63 334 deliveries, 215 (0.3%) were by patients with SCD. Among patients with SCD, the prevalence of stillbirth was 2.3%, compared with 0.8% in the general population (Table 2). In the fully adjusted model, similar to patients with SCT, those with SCD were at a higher risk of stillbirth relative to patients without SCD (aOR, 26.40; 95% CI, 2.48-280.90; *P* = .007), and SCD was the variable most strongly associated with stillbirth (Figure 3; eTable 2 in the Supplement). In addition, the stratified analysis found Black or African American patients with SCD to be at higher risk of stillbirth compared with Black or African American patients without SCD (aOR, 3.59; 95% CI, 1.41-9.12; *P* = .007) (eTable 2 in the Supplement).

### Other Variables

In addition to SCT and SCD, the fully adjusted model also found multiple gestation to be associated with an increased risk of stillbirth (aOR, 4.68; 95% CI, 3.48-6.29; *P* < .001) (Figure 3; eTable 2 in the Supplement). Patients with prior cesarean delivery had a decreased risk of stillbirth (aOR, 0.44; 95% CI, 0.23-0.82; *P* = .01), as did patients who were married at the time of delivery (aOR, 0.72; 95% CI, 0.57-0.91; *P* = .006).

A higher risk of stillbirth with multiple gestation (aOR, 3.68; 95% CI, 2.43-5.58; *P* < .001) and a lower risk of stillbirth with prior cesarean delivery (aOR, 0.30; 95% CI, 0.12-0.74; *P* = .009) were also found among Black or African Americans patients in the stratified analysis (eTable 2 in the Supplement).

## Discussion

### Sickle Cell Trait and Disease

Sickle cell trait is a condition with limited and conflicting data regarding pregnancy complications.^23^ Our retrospective cohort study evaluated a large population of affected individuals (1904 patients, 2482 deliveries) and individual-level risks associated with SCT that were also unique to each pregnancy. Our study results suggest that SCT is associated with an increased risk of stillbirth in pregnant patients . Similar results were reported in a study by Taylor et al.^11^ Importantly, the size of the affected population in our cohort study was able to bring large-scale support to the findings of Taylor et al, reinforcing the importance of SCT status in obstetric care.

Our results also suggest that patients with SCD were at increased risk of stillbirth, as reported in other studies^21,37^ and confirmed in meta-analyses.^18^ In the fully adjusted model, SCD had a stronger association than SCT with stillbirth.

These findings provide evidence that pregnant patients with SCT experience severe health complications, underscoring the importance of additional risk assessment for SCT. They also provide support for the reality that despite advances in SCD management, there continues to be a pressing need for greater systemic support for comprehensive coordinated care for patients,^32,52^ particularly during pregnancy and in the perinatal period.^37,38,39,53^

### Race and Ethnicity

The stratified analysis among only Black or African American patients did not find an association between SCT and stillbirth, a finding that disagrees with the study by Taylor et al^11^ and is consistent with the findings of Tita et al.^54^ Reasons for these differences are unclear, but we know that sickle cell studies in the US, like those of Taylor et al and Tita et al, often focus solely on African American populations, and less is known about how sickle cell manifests in other populations. Ultimately, it may be impossible to disentangle the risks due to the disease and those due to disparities associated with the disease that have resulted from longstanding inequity and stigma. Regardless, these findings speak to the importance of including racially and ethnically diverse patient populations in sickle cell research. The findings also suggest that SCT may have biological mechanisms contributing to severe clinical complications and invite a more critical examination of the assumption that SCT is not a disease state. To our knowledge, our study was the first to consider each racial and ethnic identity independently as a way to gauge the role of racism in stillbirth, opening an area of future inquiry that may otherwise have gone unnoticed.

### Multiple Gestation, Prior Cesarean Delivery, and Marital Status

Other factors found to be associated with stillbirth included multiple gestation, prior cesarean delivery, and marital status. Multiple gestation was associated with an increased risk of stillbirth in our study, which was consistent with a study by the Stillbirth Collaborative Research Network.^35^ Prior cesarean delivery was associated with a decreased risk of stillbirth, which disagrees with the increased risk reported by Reddy et al.^34^ One reason for this disparity could be that the study by Reddy et al was conducted only among singleton pregnancies, whereas our study included multiple gestation pregnancies and adjusted for multiple gestation in the regression model. Last, being married was found in our study to be associated with a decreased risk of stillbirth, which is comparable to the findings reported by SCRN that being unmarried was associated with an increased risk of stillbirth.

### Generalizability

The size and diversity of the patient population included in our study positions the findings to be generalizable to racially and ethnically diverse patient populations with singleton or multiple gestation pregnancies and who deliver at quaternary academic medical centers. Second, our study is one of the largest to investigate the association of SCT with stillbirth and also the most diverse (including both Black or African American and non–Black or African American patients in our cohort). After narrowing our cohort to only Black or African American patients with SCT, our study includes a comparable number of SCT deliveries relative to the Tita et al^54^ study and nearly 13-fold more than the Taylor et al^11^ study. More specific to stillbirths, our study included 23 SCT stillbirths, which is more than in the studies by Tita et al^54^and Taylor et al,^11^ which included 15 and 17 SCT stillbirths, respectively.

### Strengths and Limitations

The present study was strengthened by our ability to take a contemporary and innovative approach to the study of SCT through access to a sizable population of affected pregnancies, and the ability to account for individual-level risk factors and factors unique to each pregnancy. This study also had several limitations. An important limitation was that the association of SCT with stillbirth was not assessed independently of other medical comorbidities, including hypertension, preeclampsia, diabetes, obesity, etc. This territory presents an additional research opportunity that would make a critical addition to the paucity of literature examining SCT, particularly in the context of obstetric care.

Other limitations are related to the use of *ICD-9* and *ICD-10* billing codes to identify clinical conditions. Billing codes are imperfect in their ability to capture clinical conditions; for sickle cell status, there is potential to underestimate the number of patients or overestimate them because of false-positive diagnoses.^31^ However, misclassification owing to the latter is unlikely in this case because we identified patients with sickle cell over a period of approximately 7 years. Another related limitation is that we did not have data on hemoglobin variants, as available in other studies.^5,20,37^ The most common form of SCD in the US is sickle cell anemia (HbSS, homozygous), but other common variants include sickle cell hemoglobin C (HbS/C) and 2 types of sickle cell β-thalassemias (β-plus thalassemia [HbS/β^+^] and β-zero thalassemia [HbS/β^0^]).^3,55^ Including this level of granularity would help confirm sickle cell status, identify additional patients with homozygous SCD (sickle cell anemia), or identify asymptomatic patients with heterozygous sickle cell, and is a valuable path for future work.

## Conclusions

In this cohort study, SCT was associated with an increased risk of stillbirth. These results underscore the need for (1) clear pregnancy care guidance for patients with SCT and (2) systemic support for comprehensive coordinated care for SCT as well as SCD populations.

## Notes

Supplement. **eTable 1.** ICD Codes for Clinical Conditions **eTable 2.** Regression Results for Stillbirth **eFigure.** Stillbirth Rate Over Time and by Season for the Total Patient Population
