# Supplementary material for: Evaluation of Stillbirth Among Pregnant People With Sickle Cell Trait
Source: JAMA Netw Open. 2021 Nov 24;4(11):e2134274. doi: 10.1001/jamanetworkopen.2021.34274 (PMC8613600; doi:10.1001/jamanetworkopen.2021.34274)
Supplement: Supplement. — eTable 1. ICD Codes for Clinical Conditions eTable 2. Regression Results for Stillbirth eFigure. Stillbirth Rate Over Time and by Season for the Total Patient Population [file jamanetwopen-e2134274-s001.pdf]

Supplementary Online Content

Canelón SP, Butts S, Boland MR. Evaluation of stillbirth among pregnant people with sickle cell trait. *JAMA Netw Open*. 2021;4(11):e2134274. doi:10.1001/jamanetworkopen.2021.34274

eTable 1. ICD Codes for Clinical Conditions

eTable 2. Regression Results for Stillbirth

eFigure. Stillbirth Rate Over Time and by Season for the Total Patient Population

This supplementary material has been provided by the authors to give readers additional information about their work.

© 2021 Canelón SP et al. *JAMA Network Open.*

eTable 1. ICD Codes for Clinical Conditions

Diagnosis/Procedure	ICD 9	ICD 10
Sickle cell trait (diagnosis)	282.5	D57.3
Sickle cell disease (diagnosis)	282.41, 282.42, 282.60, 282.61, 282.62, 282.63, 282.64, 282.68, 282.69	D57.0, D57.00, D57.01, D57.02, D57.1, D57.20, D57.21, D57.211, D57.212, D57.219, D57.40, D57.41, D57.411, D57.412, D57.419, D57.80, D57.81, D57.811, D57.812, D57.819, 282.41, 282.42, 282.60, 282.61, 282.62, 282.63, 282.64, 282.68, 282.69
Sickle cell (pain) crisis (diagnosis)	282.42, 282.62, 282.64, 282.69	D57.0, D57.00, D57.01, D57.02, D57.21, D57.211, D57.212, D57.219, D57.41, D57.411, D57.412, D57.419, D57.81, D57.811, D57.812, D57.819
Multiple gestation (birth) (diagnosis)	651.01, 651.11, 651.21, 651.31, 651.41, 651.51, 651.61, 651.71, 651.81, 651.91, 652.61, 660.51, 662.31, 678.11, V27.2, V27.3, V27.4, V27.5, V27.6, V27.7, V31.00, V31.01, V31.1, V31.2, V32.00, V32.01, V32.1, V32.2, V33.00, V33.01, V33.1, V33.2, V34.00, V34.01, V34.1, V34.2, V35.00, V35.01, V35.1, V35.2, V36.00, V36.01, V36.1, V36.2, V37.00, V37.01, V37.1, V37.2	O30.02, O30.021, O30.022, O30.023, O30.029, O60.10X2, O60.10X3, O60.10X4, O60.10X5, O60.10X9, O60.12X2, O60.12X3, O60.12X4, O60.12X5, O60.12X9, O60.13X2, O60.13X3, O60.13X4, O60.13X5, O60.13X9, O60.14X2, O60.14X3, O60.14X4, O60.14X5, O60.14X9, O60.20X2, O60.20X3, O60.20X4, O60.20X5, O60.20X9, O60.22X2, O60.22X3, O60.22X4, O60.22X5, O60.22X9, O60.23X2, O60.23X3, O60.23X4, O60.23X5, O60.23X9, Z37.2, Z37.3, Z37.4, Z37.5, Z37.50, Z37.51, Z37.52, Z37.53, Z37.54, Z37.59, Z37.6, Z37.60, Z37.61, Z37.62, Z37.63, Z37.64, Z37.69, Z37.7, Z38.3, Z38.30, Z38.31, Z38.6, Z38.61, Z38.62, Z38.63, Z38.64, Z38.65, Z38.66, Z38.68, Z38.69
Cesarean section (diagnosis)	669.7, 669.71, 763.4, V30.01, V31.01, V32.01, V33.01, V34.01, V35.01, V36.01, V37.01, V39.01, 649.81, 649.82	O75.82, O82<br>Z38.01, Z38.31, Z38.69, Z38.66, Z38.62, Z38.64
Cesarean section (procedure)	74, 74.0, 74.1, 74.2, 74.4, 74.99	10D00Z0, 10D00Z1, 10D00Z2
Stillbirth (diagnosis)	V27.1, V27.3, V27.4, V27.7, V32.00, V32.01, V33.00, V33.01, V35.00, V35.01, V36.00, V36.01, V37.00, V37.01	Z37.1, Z37.3, Z37.4, Z37.6, Z37.60, Z37.61, Z37.62, Z37.63, Z37.64, Z37.69, Z37.7
Blood products transfusion (procedure)	99.00, 99.01, 99.02, 99.03, 99.04, 99.05, 99.06, 99.07, 99.08, 99.09	30230H0, 30230H1, 30230K0, 30230K1, 30230L0, 30230L1, 30230M0, 30230M1, 30230N0, 30230N1, 30230P0, 30230P1,

© 2021 Canelón SP et al. *JAMA Network Open.*

	30230R0, 30230R1, 30230T0, 30230T1, 30233H0, 30233H1, 30233K0, 30233K1, 30233L0, 30233L1, 30233M0, 30233M1, 30233N0, 30233N1, 30233P0, 30233P1, 30233R0, 30233R1, 30233T0, 30233T1, 30240H0, 30240H1, 30240K0, 30240K1, 30240L0, 30240L1, 30240M0, 30240M1, 30240N0, 30240N1, 30240P0, 30240P1, 30240R0, 30240R1, 30240T0, 30240T1, 30243H0, 30243H1, 30243K0, 30243K1, 30243L0, 30243L1, 30243M0, 30243M1, 30243N0, 30243N1, 30243P0, 30243P1, 30243R0, 30243R1, 30243T0, 30243T1, 30250H0, 30250H1, 30250K0, 30250K1, 30250L0, 30250L1, 30250M0, 30250M1, 30250N0, 30250N1, 30250P0, 30250P1, 30250R0, 30250R1, 30250T0, 30250T1, 30253H0, 30253H1, 30253K0, 30253K1, 30253L0, 30253L1, 30253M0, 30253M1, 30253N0, 30253N1, 30253P0, 30253P1, 30253R0, 30253R1, 30253T0, 30253T1, 30260H0, 30260H1, 30260K0, 30260K1, 30260L0, 30260L1, 30260M0, 30260M1, 30260N0, 30260N1, 30260P0, 30260P1, 30260R0, 30260R1, 30260T0, 30260T1, 30263H0, 30263H1, 30263K0, 30263K1, 30263L0, 30263L1, 30263M0, 30263M1, 30263N0, 30263N1, 30263P0, 30263P1, 30263R0, 30263R1, 30263T0, 30263T1

ICD, International Classification of Diseases

© 2021 Canelón SP et al. *JAMA Network Open.*

eTable 2. Regression Results for Stillbirth

Independent Variables	Total Population	Black/African American Pop.
Sickle Cell Status		
Sickle Cell Trait	8.94 (1.05, 75.79), p = 0.045	0.91 (0.59, 1.40), p = 0.671
Sickle Cell Disease	26.40 (2.48, 280.90), p = 0.007	3.59 (1.41, 9.12), p = 0.007
Number of Pain Crises Before Delivery	0.58 (0.17, 2.02), p = 0.395	0.72 (0.23, 2.24), p = 0.566
Number of Blood Transfusions Before Delivery	1.25 (0.66, 2.39), p = 0.497	0.59 (0.16, 2.26), p = 0.444
Prior Cesarean: Yes	0.44 (0.23, 0.82), p = 0.010	0.30 (0.12, 0.74), p = 0.009
Delivery Episode	1.06 (0.88, 1.29), p = 0.520	1.20 (0.97, 1.48), p = 0.097
Multiple Gestation (Birth) Diagnosis: Yes	4.68 (3.48, 6.29), p < 0.001	3.68 (2.43, 5.58), p < 0.001
Patient Age	1.01 (1.00, 1.03), p = 0.100	1.01 (0.99, 1.03), p = 0.270
Marital Status: Married	0.72 (0.57, 0.91), p = 0.006	0.76 (0.55, 1.05), p = 0.096
Race/Ethnicity		
Hispanic	0.88 (0.41, 1.89), p = 0.750	
Hispanic × Sickle Cell	0.18 (0.01, 3.37), p = 0.249	
Black/African American	1.20 (0.59, 2.42), p = 0.617	
Black/African American × Sickle Cell	0.11 (0.01, 0.93), p = 0.043	
White	0.55 (0.27, 1.13), p = 0.105	
White × Sickle Cell	0.32 (0.02, 6.12), p = 0.449	
Asian	0.71 (0.32, 1.58), p = 0.396	
Asian × Sickle Cell	0 (0, >100), p = 0.958	
Other or Mixed	0.80 (0.34, 1.86), p = 0.606	
Other or Mixed × Sickle Cell	0.37 (0.02, 7.18), p = 0.512	
Native Hawaiian/Pacific Islander	1.09 (0.14, 8.76), p = 0.935	
Native Hawaiian/Pacific Islander × Sickle Cell	Not Available	
American Indian/Alaskan Native	3.18 (0.67, 15.06), p = 0.144	
American Indian/Alaskan Native × Sickle Cell	Not Available	
Blood Type (reference: Blood Type O)		
Blood Type A	0.88 (0.71, 1.09), p = 0.238	0.76 (0.57, 1.00), p = 0.051
Blood Type B	0.96 (0.75, 1.23), p = 0.746	0.96 (0.72, 1.27), p = 0.764
Blood Type AB	1.17 (0.79, 1.75), p = 0.436	0.91 (0.53, 1.58), p = 0.747
Blood Type Unknown	1.14 (0.68, 1.90), p = 0.623	1.09 (0.49, 2.42), p = 0.840
Rhesus (Rh) Factor: Negative	1.15 (0.84, 1.58), p = 0.380	1.21 (0.80, 1.85), p = 0.364
Year (reference: 2010)		
2011	1.17 (0.85, 1.60), p = 0.337	1.04 (0.72, 1.51), p = 0.832
2012	0.86 (0.61, 1.21), p = 0.387	0.72 (0.47, 1.09), p = 0.122
2013	0.82 (0.57, 1.17), p = 0.265	0.64 (0.41, 1), p = 0.051
2014	0.88 (0.63, 1.25), p = 0.487	0.86 (0.57, 1.29), p = 0.468
2015	0.90 (0.63, 1.27), p = 0.530	0.0 (0.53, 1.23), p = 0.312
2016	0.97 (0.69, 1.37), p = 0.882	0.92 (0.60, 1.39), p = 0.682
2017	1.13 (0.76, 1.69), p = 0.541	1.02 (0.62, 1.69), p = 0.932

Race/ethnicity categories are "Non-Hispanic" unless otherwise specified

© 2021 Canelón SP et al. *JAMA Network Open.*

eFigure. Stillbirth Rate Over Time and by Season for the Total Patient Population

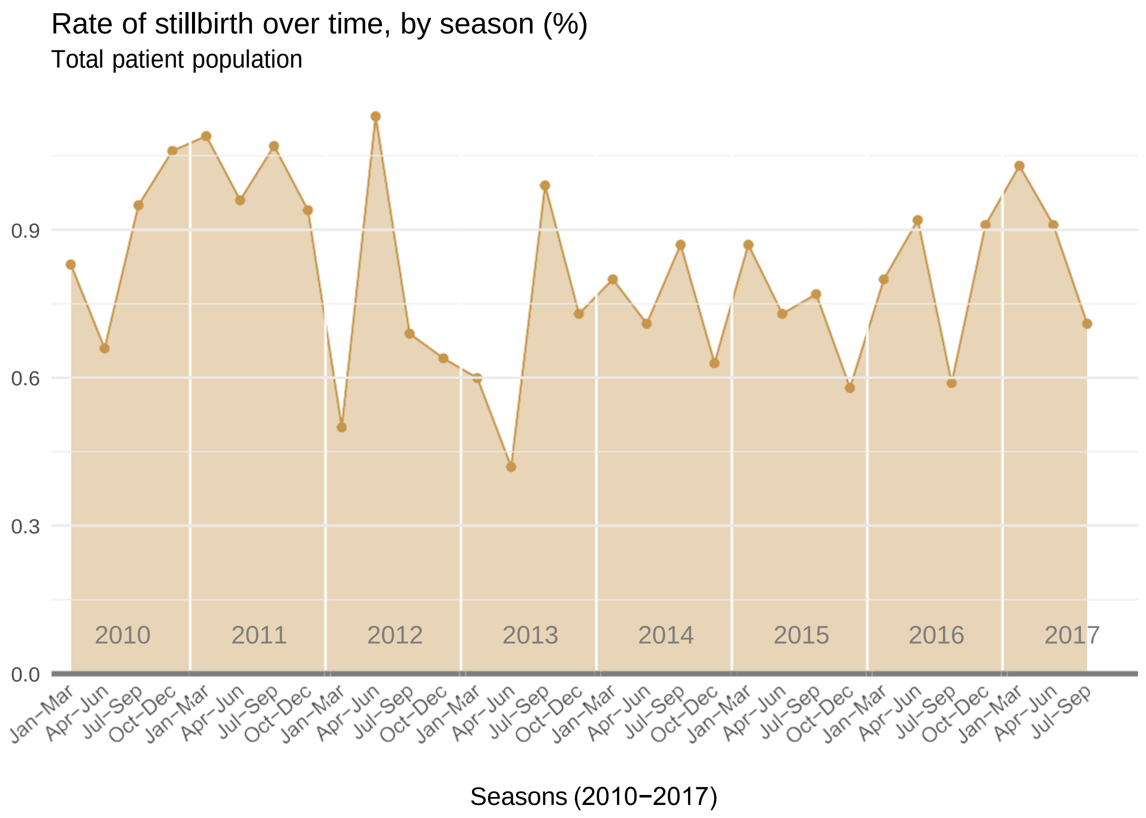

Presents stillbirth rates from January-March 2010 to July-September 2017 as an area graph. Stillbirth rates vary from 0.4% in April-June 2013 to 1.1% in April-June 2012, and there is no distinguishable trend over time.

© 2021 Canelón SP et al. *JAMA Network Open.*
